# *Salmonella bongori* 48:z_35_:– in Migratory Birds, Italy

**DOI:** 10.3201/eid1503.080039

**Published:** 2009-03

**Authors:** Maria Foti, Antonio Daidone, Aurora Aleo, Alessia Pizzimenti, Cristina Giacopello, Caterina Mammina

**Affiliations:** University of Messina, Messina, Italy (M. Foti, A. Daidone, A. Pizzimenti, C. Giacopello); University of Palermo, Palermo, Italy (A. Aleo, C. Mammina)

**Keywords:** Salmonella bongori, birds, Italy, letter

**To the Editor**: Serovars of *Salmonella,* other than subspecies *enterica,* are primarily associated with cold-blooded animals and infrequently colonize the intestines of warm-blooded animals. Strains of *S. bongori*, in particular, are rarely isolated from human patients (*1*,*2*). However, during 1984–2004, 27 strains of *S. bongori* with the antigenic formula 48:z_35_:– were identified from 2 epidemic clusters in Italy (Messina, October 1984–May 1985, and Palermo, July–October 1998), and from sporadic cases of acute enteritis that occurred in several cities in Sicily. Almost all cases involved children 1 month to 3 years of age (*3*). By 2006, 8 additional isolates of the same serovar had been collected in southern Italy from the following sources: 1) a healthy human carrier; 2) 3 warm-blooded animals (2 apparently healthy pigeons and a dog with diarrhea); 3) 2 food products (soft cheese and the shell of a hen’s egg); and 4) urban wastewater (*3*).

No cases of human infections caused by *S. bongori* 48:z_35_:– have been reported in countries other than Italy. Except for the original strain isolated from a lizard in Chad in 1966 (*4*), the only recorded isolates of this serovar are 4 isolates from foodstuffs recovered in England in 1985 (M.Y. Popoff, pers. comm.).

Pulsed-field gel electrophoresis (PFGE) analysis of *Xba*I-digested DNA of the 35 isolates obtained from Sicily showed identical or similar profiles that differed from each other by 2–5 bands (>79% similarity), except for the profile obtained from the soft cheese isolate (<40% similarity) (*3*). Thus, all but one of the isolates could be considered closely or at least likely related to the oldest profile characterizing the putative ancestor clone.

Until now, the rare *S. bongori* 48:z_35_:–, an apparent epidemiologic peculiarity of Sicily, could not have been traced to a well-defined source. Indeed, in past years, many infrequently isolated and new serovars of *Salmonella* have been identified in Sicily from wild reptiles, but *S. bongori* 48:z_35_:– has never been isolated from these animals (*5*). Moreover, whether pigeons that live in urban areas have epidemiologic importance as sources of infection is questionable, because of the apparently exclusive infection of infants in the first months of life. The role of migratory birds has not previously been assessed.

To determine the prevalence of birdborne pathogens in the migratory bird fauna of the Mediterranean basin, we conducted a study in October 2006, during ringing (banding) activity at the station of the University of Palermo on Ustica, a small island in the Tyrrhenian Sea (38°42′N, 13°11′E) near the northern coast of Sicily. Apparently healthy birds trapped by mist nets during active migration were sampled. Fresh fecal samples or cloacal swabs from 239 birds belonging to the orders Passeriformes, Gruiformes, and Caprimulgiformes were analyzed to determine whether birds were colonized with *Salmonella*. Routine procedures for isolation of *Salmonella* spp. were used. Colonies with morphologic characteristics of *Salmonella* spp. were fully identified by standard biochemical and serologic testing.

Two isolates, from 2 blackcaps (*Sylvia atricapilla*), were identified as *Salmonella* spp. Morphologic features of the external flight apparatus and weight clearly indicated that the 2 individuals belonged to the migratory subspecies of blackcap (*6*).

The *Salmonella* isolates were characterized by serotyping at the Centre for Enteric Pathogens of Southern Italy, University of Palermo, as *S. bongori* 48:z35:–. Molecular typing by PFGE after digestion of DNA by *Xba*I showed a banding pattern similar to that of all previously identified *S. bongori* 48:z35:–. In particular, the DNA restriction pattern proved to be indistinguishable from patterns of the human isolates belonging to the epidemic clusters and of the pigeon isolates (*3*).

Previous studies have documented that carriage of *Salmonella* spp. by apparently healthy migrating birds is infrequent, although some isolates have been recovered (*7*,*8*). Prevalence has been shown to be higher during breeding season and during wintering in some urban-associated bird species, especially in those that feed on refuse, such as corvids and gulls (*9*). However, to our knowledge, *S. bongori* in migratory birds has not previously been reported.

Our findings suggest that passerine migratory birds may play a role in the introduction or persistence of *S. bongori* 48:z35 in southern Italy. The bacteria also have the potential for gaining access to the food chain, as confirmed by their presence in the shell of hen eggs. Moreover, a bird-environment-food network could perpetuate a reservoir of *S. bongori* 48:z35:–. Most small passerine migratory birds, including blackcaps, do not share a niche with humans and are most likely to be found in rural habitats (*10*). However, some species are developing an increasing ability to live in urban and suburban environments, especially where winter feeding by humans attracts birds, forcing changes in the species balance. Because of their ability to fly through long distances during annual migrations, wild birds could also play a role in the epidemiology of zoonoses. Thus, risk assessment of *Salmonella* carriage in wild birds warrants further investigation.
